# Low transcriptomic of PTPRCv1 and CD3E is an independent predictor of mortality in HIV and tuberculosis co-infected patient

**DOI:** 10.1038/s41598-022-14305-8

**Published:** 2022-06-16

**Authors:** Gebremedhin Gebremicael, Atsbeha Gebreegziabxier, Desta Kassa

**Affiliations:** Ethiopian Public Health Institute (EPHI), P.O.Box: 1242, Addis Ababa, Ethiopia

**Keywords:** Immunology, Microbiology

## Abstract

A comprehensive assessment of immunological profiles during HIV-TB co-infection is essential to predict mortality, and facilitate the development of effective diagnostic assays, therapeutic agents, and vaccines. Expression levels of 105 immune-related genes were measured at enrolment and 6th month follow-up from 9 deceased HIV and TB coinfected patients who died between 3 and 7th months follow-up and at enrolment, 6th and 18th month from 18 survived matched controls groups for 2 years. Focused gene expression profiling was assessed from peripheral whole blood using a dual-color Reverse-Transcription Multiplex Ligation-dependent Probe Amplification assay. Eleven of the 105 selected genes were differentially expressed between deceased individuals and survivor-matched controls at baseline. At baseline, *IL4δ2* was significantly more highly expressed in the deceased group than survivor matched controls, whereas *CD3E, IL7R, PTPRCv1, CCL4, GNLY, BCL2, CCL5, NOD1, TLR3,* and *NLRP13* had significantly lower expression levels in the deceased group compared to survivor matched controls. At baseline, a non-parametric receiver operator characteristic curve was conducted to determine the prediction of mortality of single genes identified *CCL5, PTPRCv1*, *CD3E, and IL7R* with Area under the Curve of 0.86, 0.86, 0.86, and 0.85 respectively. The expression of these genes in the survived control was increased at the end of TB treatment from that at baseline, while decreased in the deceased group. The expression of *PTPRCv1*, *CD3E, CCL5,* and *IL7R* host genes in peripheral blood of patients with TB-HIV coinfected can potentially be used as a predictor of mortality in the Ethiopian setting. Anti-TB treatment might be less likely to restore gene expression in the level expression of the deceased group. Therefore, other new therapeutics that can restore these genes (PTPRCv1, CD3E, IL7R, and CCL5) in the deceased groups at baseline might be needed to save lives.

## Introduction

Tuberculosis (TB), caused by *Mycobacterium tuberculosis* (MTB), remains to be the major global health threat. According to the World Health Organization (WHO), there were 10.0 million (range 8.9–11.0 million) new TB patients in 2019^1^. Globally, HIV-related tuberculosis comprises, 8.2% of new tuberculosis cases but contributes a disproportionate around 15% of tuberculosis deaths in 2019^1^. WHO recommended developing an effective diagnostic and treatment for latent TB infection to achieve the reduction of death from MTB by 35% from 2015 to 2020^2^. The cumulative reduction between 2015 and 2019 was only 14%, less than half of the first milestone of the End TB Strategy^1^. After adjusting confounding factors (age, sex, residence, WHO stage, and body weight), People living with HIV and TB co-infection had 40% higher mortality than those without TB^3^. Moreover, HIV and TB co-infection have an adjusted 2.08 times higher risk of mortality compared to TB patients^4^. The underlying causes of mortality are poorly understood. In HIV-related tuberculosis, early mortality has been associated with proxy for severe immunosuppression^5^, increases immune activation^6–8^, and failure to recover cellular immune responses to *Mycobacterium tuberculosis*^9^, lack of effective immunity at baseline^10^.

Prediction of mortality during HIV and TB co-infection is a major challenge. Furthermore, the currently available diagnostic tools cannot predict mortality during HIV and TB co-infection, and cannot distinguish which patients can survive or die. The study of the immune response and assessment of factors that contribute to mortality may allow for the development of better diagnostics, therapeutics, treatment monitoring tools. Importantly, the association of host gene expression with the risk of mortality in HIV and TB co-infected individuals is not adequately investigated. Therefore, our study was focused on host gene expression for assessing the mortality risk of TB-HIV co-infected individuals. Therefore, we aimed to assess host gene expression in whole blood that can be predict mortality during HIV-TB co-infection using focused gene expression profiling by dual-colour Reverse-Transcription Multiplex Ligation-dependent Probe Amplification (dcRT-MLPA).

## Materials and methods

### Ethics statement

All study participants requested if provided written, informed consent at enrollment. This study obtained ethical clearance from the Scientific and Ethics Research Office of the Ethiopian Public Health Research Institute (Ref: E.H.N.R.I. 6.13/01) and the London School of Hygiene & Tropical Medicine Ethics Review Committee (Ref: 7174). All study procedures were conducted according to the Declaration of Helsinki.

### Study design and population

**Figure 1:**
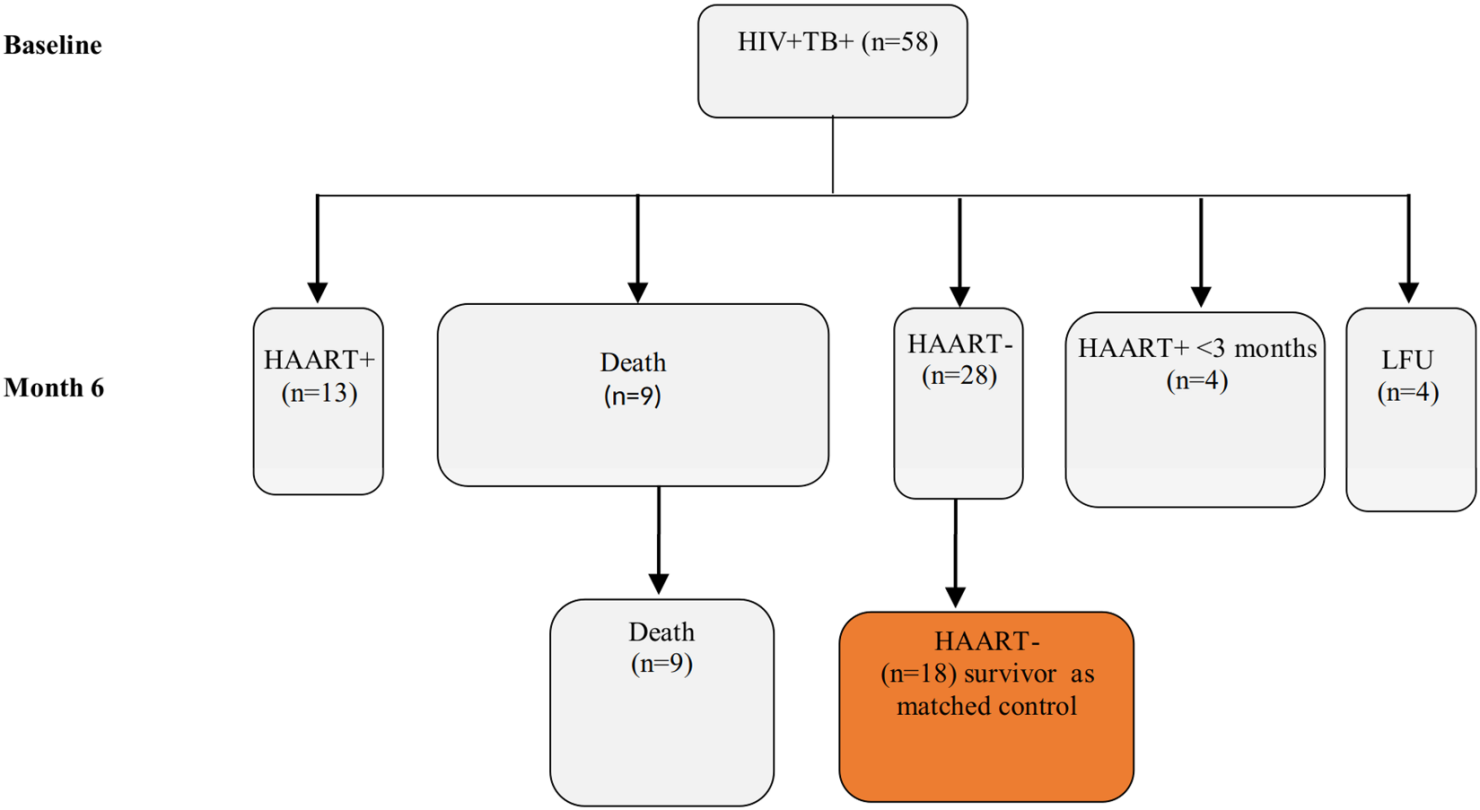
Flow diagram of the cohort. At enrollment 58 HIV + TB+ subjects were enrolled: All HIV + TB+ patients were ATT treated at baseline. *LFU* Lost during follow up, *HAART+* Subjects that had received HAART treatment for at least 3 months at 6th month follow up *HAART−* Subjects that had not received HAART treatment at the indicated time point. All death group was HAART− until the death date.

This matched case–control and an observational cohort study was part of the previous study^11^. The study participants were 9 active TB-HIV infected patients (HIV + TB +) who died between 3 and 7 months, and 18 HIV + TB + survived patients which were followed at 6th, 18th and 24 months (Fig. 1). All of the deceased groups and survived controls were not HAART treated. Whole blood was collected from the 5 of 9 deceased groups before they deceased. For each deceased individuals, two matched controls that remained survived during 2 years of follow-up time were selected and matched by age at enrolment, sex (≤ 18 years, 19–25 years, 26–35 years, or ≥ 36 years), and, year of enrolment^12^, CD4+ count and viral load. All cases and controls were treated for TB treatment at recruitment time according to the national guideline^13^.

#### Diagnostic assessment

HIV status was determined using the Determine HIV-1/2 screening test (Abbott Laboratories, Tokyo, Japan), the Capilus HIV1/2 as a confirmatory test (TrinityBiotec, Wicklow, Ireland), and the Unigold HIV-1/2 recombinant test (TrinityBiotec, Ireland) as a tiebreaker^13^. The CD4+ T-cell count was determined by flow cytometry, using the FACSCalibur platform (Becton Dickinson, USA), while the plasma HIV RNA load was determined using the NucliSensEasyQ NASBA diagnostic kit (OrganonTeknica, the Netherlands). A diagnosis of active tuberculosis was based on both clinical and bacteriological parameters. At least 2 sputum smears (a spot sample and an early morning sample) were required to be positive by microscopy for acid-fast bacilli, using the ZiehlNeelsen staining method (independent of the presence/absence of clinical parameters).

#### Laboratory procedure

##### RNA extraction

A total of 2.5 mL of venous blood was collected into Paxgene Blood RNA tubes (PreAnalytiX, Qiagen, Germany). RNA was extracted spin column-based using the Paxgene RNA extraction kit (PreAnalytiX, Qiagen) according to the manufacturer’s instructions. After the Paxgene tubes were briefly centrifuged at 4000 rpm for 10 min, the pellet of cells was lysed using lysing Buffer (Buffer BR1). The unwanted protein in the lysed pellet was removed using proteinase K. Ethanol-precipitated nucleic acids were loaded onto a spin column. Unwanted DNA was digested using RNase-free DNase (Qiagen). Finally, purified RNA was eluted with RNase-free elution buffer (BR5 buffer) and quantified using a NanoDrop 2000 Spectrophotometer (Thermo Fisher Scientific, Wilmington, USA). The ratio of absorbance at 260 nm and 280 nm (A260/A280) ranging from 1.8 to 2.2 was considered pure RNA. Moreover, the samples with the ratio absorbance range of below 1.70 or above 2.3 were excluded from further analyses due to those samples do have not enough RNA for MLPA analysis.

##### dcRT-MLPA

dcRT-MLPA was performed as described in detail previously^14^. Briefly, for each target‐specific sequence, a specific reverse transcription (RT) primer was designed located immediately downstream of the left and right-hand half‐probe target sequence. Complementary DNA (cDNA) was generated from 125 ng RNA by incubation at 37 °C for 15 min using an RT primer mix and Moloney murine leukemia virus (MMLV) reverse transcriptase (Promega, USA). Reverse transcriptase activity was inactivated by heating at 98 °C for 2 min and cDNA was incubated overnight at 60 °C with a mixture of customized left and right-hand half‐probes to hybridize with the target cDNA. Annealed half‐probes were ligated using ligase-65 enzyme and subsequently amplified by PCR (33 cycles of 30 s at 95 °C, 30 s at 58 °C, and 60 s at 72 °C, followed by 1 cycle of 20 min at 72 °C). Primers and probes were from Sigma-Aldrich Chemie (Zwijndrecht, The Netherlands) and MLPA reagents from MRC-Holland (Amsterdam, The Netherlands). PCR amplification products were 1:10 diluted in HiDi formamide‐containing 400HD ROX size standard, denatured at 95 °C for 5 min, cooled on ice, and analyzed on an Applied Biosystems 3730 capillary sequencer in GeneScan mode (Base Clear, Leiden, The Netherlands).

Trace data were analyzed using GeneMapper software 5 package (Applied Biosystems). The areas of each assigned peak (in arbitrary units) were exported for further analysis in Microsoft Excel spreadsheet software. The data were normalized against each of the four reference genes (GAPDH, B2M, GUSB, or ABR). The GAPDH normalized data was the best to use for downstream analysis since this gene was nicely expressed in almost all samples. Signals below the threshold value for noise cutoff in GeneMapper (log2 transformed peak area 7.64) were assigned the threshold value for noise cutoff. Finally, the normalized data were log2 transformed for statistical analysis.

RT primers and half-probes were designed by Leiden University Medical Centre (LUMC, Leiden, The Netherlands)^15,16^ and comprise sequences for 4 housekeeping genes and 105 selected genes to profile the innate and adaptive immune response^14^. These selected genes were related to active TB disease or protection against disease identified as described in previous studies in the literature.

### Statistical analysis

A non-parametric two-tailed Wilcoxon rank-sum (Mann–Whitney) test was used to compare two unpaired data sets after assessment of the normality of the data using the Kolmogorov Smirnov test. Non-parametric Receiver Operator Characteristic (ROC) curves were used to determine the potential predictor of mortality in HIV + TB+ patients. Spearman rank correlation was used to measure the degree of association between mortality and single host gene expression. The statistical significance level used was P < 0.05 and all P values are two-tailed. All data analysis was performed using Inter cooled STATA version 16.0 (College Station, Texas, USA).

## Results

### Characteristics of the study population at baseline

Nine HIV + TB+ who died between 3 and 7 months, and 18 matched control HIV + TB+ survived patients which were followed at 6th, 18th and 24 months were included in this matched case–control and an observational cohort study. Baseline demographic, clinical, and laboratory data for each study group are shown in Supplementary Table 1. The mean age (± SD) of deceased groups, and matched controls was 31.9 ± 8.9 and 32.4 ± 9.6 years, respectively, and 33.3% of both the groups were female. Statistically significant differences between cases and matched controls were not observed in the proportion of patients with malnutrition (defined as a body mass index [calculated as the weight in kilograms divided by the height in meters squared] of < 18.50)^17^, CD4+ T-cell count, and HIV-1 RNA load (Supplementary Table 1).

### Gene expression profiles in deceased groups and survived matched controls

Host gene expression in whole blood from 9 deceased groups and 18 survivor matched controls were analysed by dcRT-MLPA using probe sets for 105 selected gene profiles (targeting innate and adaptive immune responses genes) (Supplementary Table 2). Eleven of the 105 selected genes were differentially expressed between deceased groups and survivor-matched controls at baseline (Table 1). Thus, one gene (*IL4δ2*) was significantly higher expressed in deceased groups than survivor matched controls, whereas 10 genes (*CD3E, IL7R, PTPRCv1, CCL4, GNLY, BCL2, CCL5, NOD1, TLR3,* and *NLRP13*) had significantly lower expression in deceased groups compared to survivor matched controls.

**Table 1:** Gene expression profiles in deceased groups and survived controls.

Gene symbol	HIV + TB+ deceased (n = 9)	HIV + TB+ survivor as control (n = 18)	P-value
**T cell subset markers**			
CD3E	12.27 (10.25–12.27)	13.21 (12.68–13.49)	**0.0033**
IL7R	9.98 (9.62–10.58)	12.02 (11.59–12.25)	**0.0039**
PTPRCv1	9.20 (8.99–9.95)	10.50 (10.03–11.62)	**0.0028**
**Th2/Treg associated genes**			
IL4δ2	12.15 (9.78–13.17)	7.64 (7.64–11.20)	*0.0200*
CCL4	9.38 (9.16–9.72)	10.07 (9.39–10.25)	**0.0492**
**Cytotoxicity genes**			
GNLY	12.01 (11.26–13.42)	13.88 (12.81–14.28)	**0.0333**
**Apoptosis/survival**			
BCL2	8.27 (7.64–8.97)	9.54 (8.92–9.89)	**0.0458**
**Myeloid associated genes**			
CCL5	13.93 (13.14–14.37)	14.70 (14.36–14.83)	**0.0033**
**Pattern recognition receptors**			
NOD1	7.79 (7.64–8.02)	8.32 (7.96–8.52)	**0.0183**
TLR3	9.63 (9.45–10.12)	10.40 (10.21–10.82)	**0.0380**
**Inflammasome components**			
NLRP13	7.64 (7.64–7.64)	7.64 (7.64–9.32)	**0.0177**

Median (interquartile range) gene expression values (peak areas normalized for GAPDH and log2-transformed) are shown at the baseline of the death groups and controls, and significant differences between the death groups and controls were determined using the Wilcoxon Mann–Whitney test. In italics: genes are indicated that were more highly expressed in the test group compared to the reference/control group. In bold: genes are indicated that had lower expression in the test group compared to the reference/control group. Only genes whose expression level significantly differed between the two study groups are listed.

**Figure 2:**
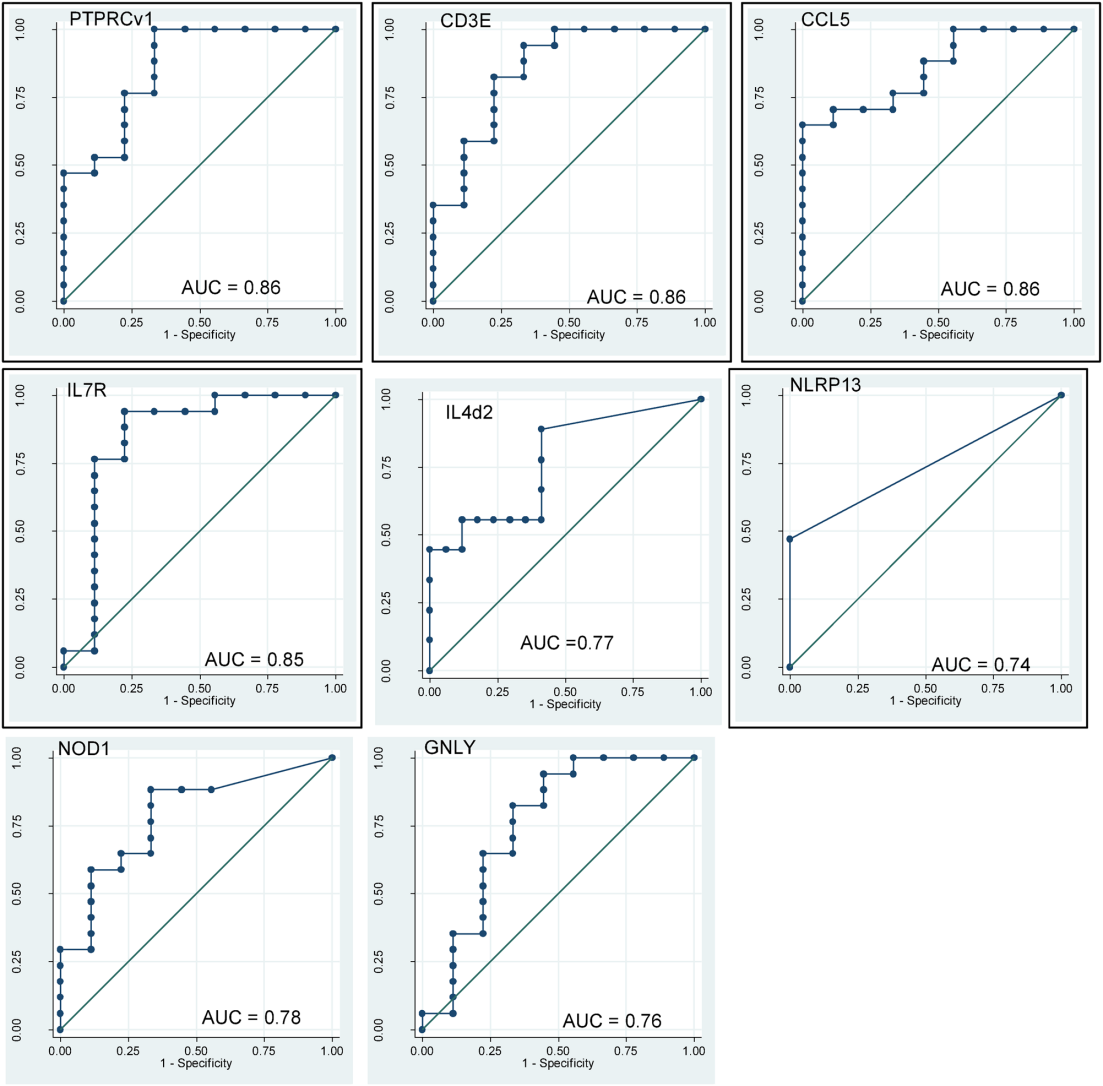
Single host genes with discriminatory power to classify death groups from survived groups. Receiver operator characteristics (ROC) curves showing the accuracies of individual genes in discriminating death groups versus survival groups. *AUC* Area under the curve.

**Figure 3:**
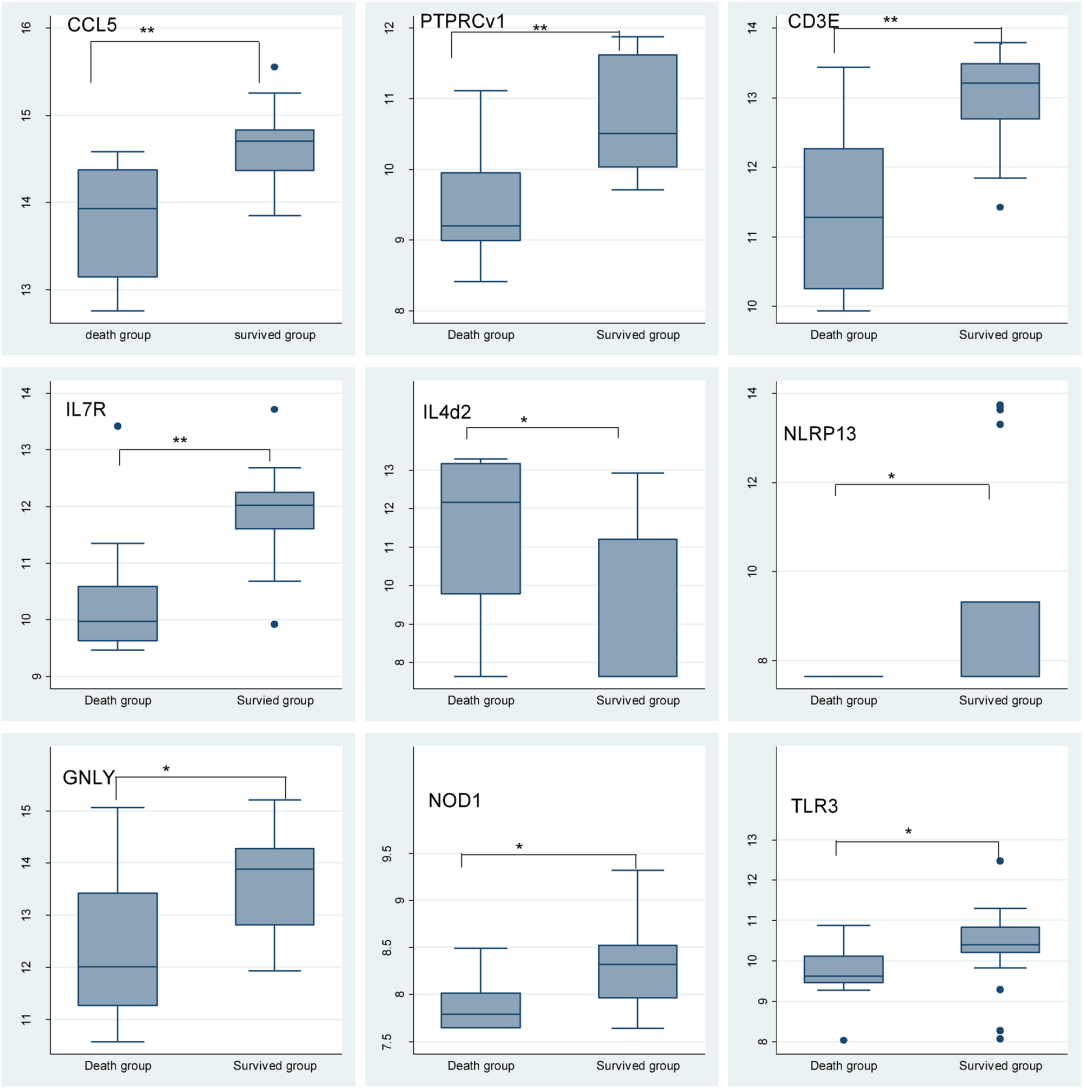
Gene expression profiles of signature genes. Median gene expression levels (peak areas normalized to GAPDH and log2-transformed) of the indicated genes are shown as box-and-whisker plots (5–95 percentiles). Significant differences between study groups were determined using Wilcoxon Mann–Whitney test. Shown are individual genes that were found to have the best discriminatory power to distinguish between death groups versus survival groups. (*P-value ≤ 0.05, **P-value ≤ 0.01, ***P-value ≤ 0.001, ****P-value ≤ 0.0001).

At baseline, non-parametric receiver operator characteristic (ROC) curves was analysed to determine the prediction of mortality of single genes identified *CCL5, PTPRCv1*, *CD3E, IL7R*, *NOD1*, *IL4δ2,* and *GNLY* with Area Under the Curve (AUCs) of 0.86, 0.86, 0.86, 0.85, 0.78, 0.77 and 0.76 respectively, indicating those genes with the most powerful classifying potential biomarker for risk of mortality among survivors of HIV + TB+ (Fig. 2). The gene expression profiles of these signature genes are displayed in Fig. 3.

**Figure 4:**
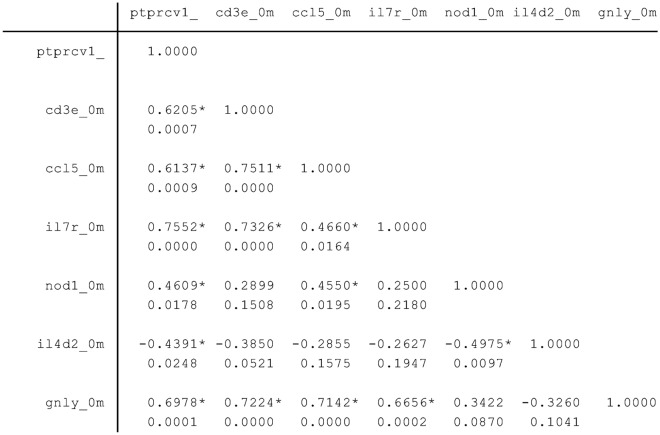
The relationship among the expression of genes. Spearman analysis was done to look the relationship among the expression of genes. Upper number indicates the degree of relation and lower number indicates P-value. – Sign indicates negative relation and * indicates had relation between the host gene expression.

After performing the spearman analysis to look at the relationship among the expression of genes, the gene expression of PTPRCv1 had a positive correlation with 5 gene expressions (CD3E, CCL5, IL7R, NOD1, and GNLY), but a negative correlation with IL4d2 (Fig. 4). However, the gene expression of CD3E and IL7R did not correlate with the expression of NOD1 and IL4d2.

**Figure 5:**
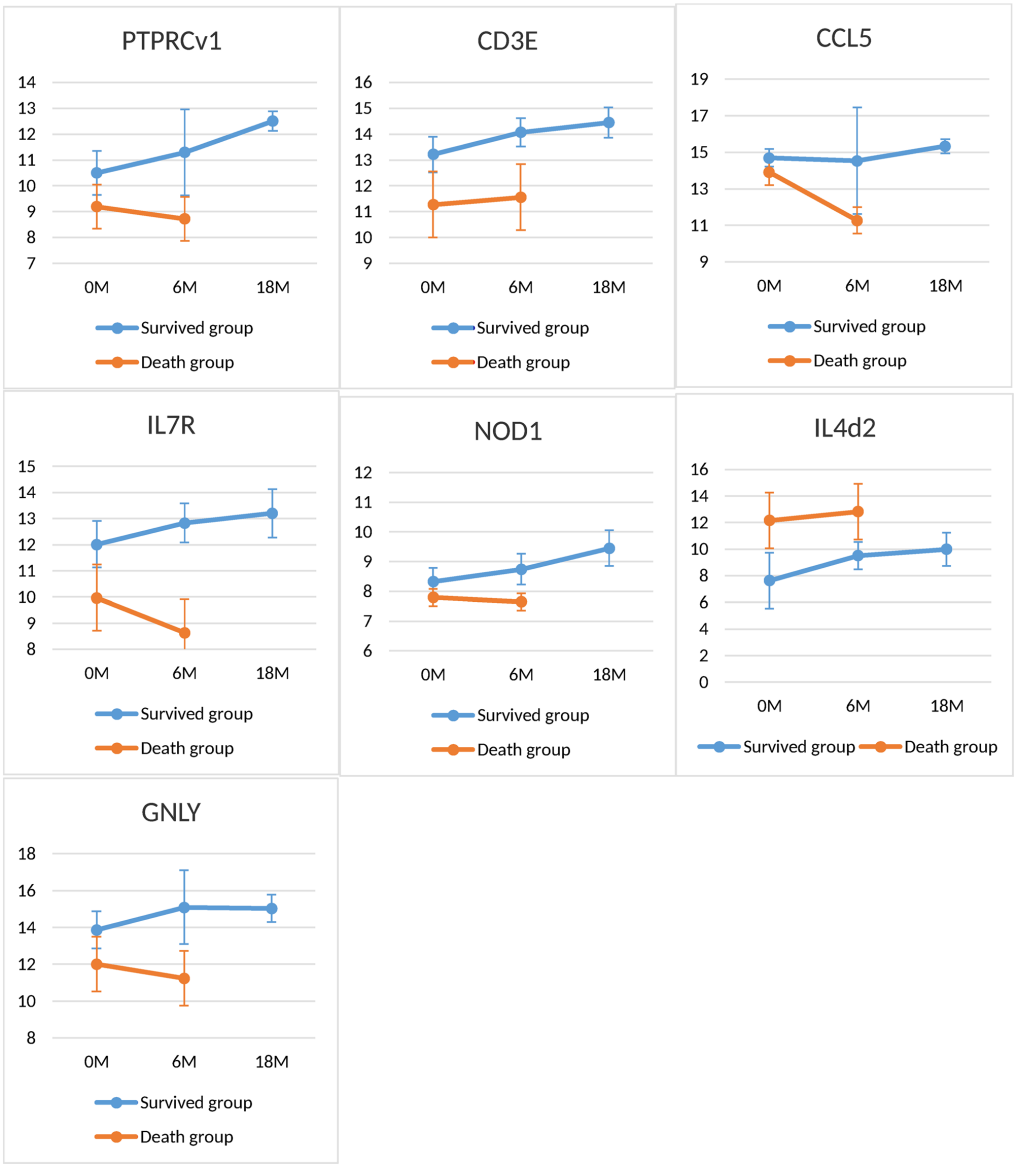
Anti-TB treatment response transcriptomic profiles. Median gene expression levels (peak areas normalized to *GAPDH* and log2-transformed) and standard deviation are shown of the indicated genes at baseline (0 M) and 6 months after treatment initiation (6 M) and (18 M) of study groups.

The host gene expressions at the follow-up time (6th and 18th months) in the survived matched control were increased compared to the expression at baseline (Fig. 5). However, host gene expression in the deceased groups was decreased or plane compared the expression at baseline level with that at TB treatment complement time. Therefore, anti-TB treatment may not have any impact on the gene expression in HIV + TB+ patients with severe diseases (at the level of the deceased group).

## Discussion

Assessing immunological profiles predictors to mortality in HIV + TB+ co-infection patients is crucial for the development of effective assays, the invention of novel therapeutic targets, and permitting suitable risk stratification to target new interventions correctly, and aiding monitoring of treatment responses. The findings of the study showed that *IL4δ2* was significantly high expressed in the deceased group than survivor matched controls, whereas *CD3E, IL7R, PTPRCv1, CCL4, GNLY, BCL2, CCL5, NOD1, TLR3,* and *NLRP13* had significantly lower expression levels in deceased group compared to survivor matched controls at baseline. However, after conducting ROC curves analysis, 8 single genes (*CCL5, PTPRCv1*, *CD3E, IL7R*, *NOD1*, IL-4δ2, and *GNLY*) with AUCs ≥ 75 could accurately differentiate the groups of HIV + TB+ co-infected individuals at baseline. This result indicating these interrelating biomarkers are strongly associated with HIV + TB+ co-infected related mortality.

The lower expression of T-cell associated genes (PTPRCv1, CD3E, and IL7R) in these deceased groups at baseline may reflect reduced responsiveness of TCR signaling which may represent an HIV-induced evasion mechanism in individuals with weak immune systems^18–20^. Given the fact that HIV infection accelerates decreasing of the expression of all investigated T cell subsets which leads to HIV progression. However, our result shows the lower expression of PTPRCv1 cells in deceased groups was associated with an increased risk of dying which is similar to the previous report^21^. This lower expression may be related to the depletion of naive T cells^22,23^, and a relative expansion of immature and activated CD4+ cells^24,25^. Lower gene expression of CD3E in deceased groups suggests a lack of pre-TCR-associated intrinsic kinase activity which may play a critical role in transmitting a signal across T cell membrane^26^. A previous study demonstrated that low CD3E expression was a predictor of poor survival^27^. This may be correlated with the infiltration levels of CD8+ T cells to the infection site that leads to deletion of T-cells and with the increase of pathological stage^28^. Lower expression of IL7R was also reported in AIDS patients elsewhere previously^29^. These findings show impaired T-cell functions and immune failure described in HIV + TB+ co-infected patients that can predict mortality. The function of IL-7R has improved the functionality of tolerized CD8 T cells their ignorance of self-antigens and promoted CD8 T cell cytotoxicity to self-antigens which protects autoimmune^30^.

Our finding shows that the gene expression of CCL5 (Myeloid-associated gene) was amongst the strongest differentially expressed genes between deceased groups and survived matched controls, with potential discriminatory power between deceased groups and survived matched controls. This is consistent with the expression profiles of soluble CCL5/RANTES from plasma^8^. T cells preferentially used CCR5 interactions with ligands (CCL5 and CCL4) for early migration into MTB-infected lungs and granuloma formation^31^, which indicates CCL5 contributes to early protective, and MTB-specific immunity^32^, but may also important role in immunopathogenesis during TB^33^.

The host gene expressions at the follow-up time (6th and 18th months) in the survived matched control were increased compared to the expression at baseline (Fig. 5). However, host gene expression in the deceased groups was decreased or plane compared the expression at baseline level with that at TB treatment complement time. Therefore, anti-TB treatment may not have any impact on the gene expression in HIV + TB+ patients with severe diseases (at the level of the deceased group).

The survived matched control had increased host gene expression over the follow-up time (at 6th and 18th months) from baseline (Fig. 5), which is similar to our previous finding ‘most gene expressions were normalized at the end of anti-TB treatment of TB patients to levels observed in latent TB infected and uninfected control subjects during HIV infection”^11^. This finding shows clearing of the TB bacilli from the infection body and restoring gene expression due to anti-TB treatment. However, anti-TB treatment may be less likely to clear off the TB bacilli and restore gene expression in these deceased groups. In our previous study, at baseline, there was no giant distinction between host gene expression of HAART eligible and ineligible HIV + TB+ groups^11^. Moreover, HAART has no impact on the level of gene expression in HIV + TB+ patients with severe diseases (at the level of the deceased group). However, the superiority of mortality amongst HAART ineligible groups was better than in eligible groups^34^. Therefore, other therapies that can restore these genes (PTPRCv1, CD3E, IL7R, and CCL5) might be needed to save lives.

Our finding also shows low gene expressions of NOD1 and NLRP13 in deceased HIV + TB+ groups compared to survived groups, but less likely to predict the mortality. Mekonnen and his colleagues demonstrated that *NOD1* identified with novel genetic associations with TB in Ethiopian populations^35^. NOD1 plays important role in the detection of bacteria and the production of proinflammatory molecules, given that both signaling pathways lead to NF-κB and MAPK activation^36^. NOD1 deficiency mice show increased susceptibility to lung infection, which is associated with reduced neutrophil recruitment to the lungs^37^, and associated with the development of inflammatory disorders^38^. In contrast to the other gene expression, the gene expression of IL-4δ2 was higher in the deceased HIV + TB+ groups compared to survival matched controls. Dheda et al. demonstrated that IL-4δ2 was expressed higher in TB patients compared to controls and in HIV infected TB patients compared to HIV uninfected patients which correlated with disease severity^39,40^. Therefore, disease severity may have a direct implications for mortality. However, IL4δ2 had a wide 95% CI range and less than 0.65 lower limit of AUC [AUC (95% CI) 0.77 (0.57–0.97)]. Therefore, IL-4δ2 may have less probable to predict mortality.

In conclusion, the expression of PTPRCv1, CD3E, CCL5, and IL7R in peripheral blood can predict mortality in HIV + TB+ co-infected individuals. Anti-TB treatment might be less likely to restore gene expression in the level expression of the deceased group. Therefore, other new therapeutics or vaccine that can restore these genes (PTPRCv1, CD3E, IL7R, and CCL5) in HIV + TB+ patients with severe diseases at baseline might be needed to save lives. In the future, the identified biomarkers could be applied to facilitate the development of effective assays for prediction of mortality in HIV + TB+ patients, the discovery of new drugs and vaccines that could restoring the gene expression of these genes (PTPRCv1, CD3E, CCL5, and IL7R), and aid monitoring of treatment responses. Future work could determine to completely characterize these signatures in different populations and larger study populations.

## Supplementary Information

Supplementary Tables.

## Data Availability

All necessary data generated or analyzed during this study are included in this article.

## References

[1] World Health Organization. Global tuberculosis report 2020. World Health Organization. https://apps.who.int/iris/handle/10665/336069. License: CC BY-NC-SA 3.0 IGO. (2020).

[2] Executive Board, 134. Global strategy and targets for tuberculosis prevention, care and control after 2015: Report by the Secretariat. https://apps.who.int/iris/handle/10665/172828. (2014).

[3] Mollel EW, Todd J, Mahande MJ, Msuya SE (2020). Effect of tuberculosis infection on mortality of HIV-infected patients in Northern Tanzania. Trop. Med. Health.

[4] Stijnberg D (2019). Factors associated with mortality in persons co-infected with tuberculosis and HIV in Suriname: A retrospective cohort study. Rev. Panam. Salud. Publica.

[5] Straetemans M, Bierrenbach AL, Nagelkerke N, Glaziou P, van der Werf MJ (2010). The effect of tuberculosis on mortality in HIV positive people: A meta-analysis. PLoS One.

[6] Sattler FR (2018). Biomarkers associated with death after initiating treatment for tuberculosis and HIV in patients with very low CD(4) cells. Pathog. Immun..

[7] Ravimohan S (2015). Immunological profiling of tuberculosis-associated immune reconstitution inflammatory syndrome and non-immune reconstitution inflammatory syndrome death in HIV-infected adults with pulmonary tuberculosis starting antiretroviral therapy: A prospective observational cohort study. Lancet Infect. Dis..

[8] Schutz C (2019). Clinical, microbiologic, and immunologic determinants of mortality in hospitalized patients with HIV-associated tuberculosis: A prospective cohort study. PLoS Med..

[9] Ravimohan S (2013). Early immunologic failure is associated with early mortality among advanced HIV-infected adults initiating antiretroviral therapy with active tuberculosis. J. Infect. Dis..

[10] Barry CE (2009). The spectrum of latent tuberculosis: Rethinking the biology and intervention strategies. Nat. Rev. Microbiol..

[11] Gebremicael G (2018). Host gene expression kinetics during treatment of tuberculosis in HIV-coinfected individuals is independent of highly active antiretroviral therapy. J. Infect. Dis..

[12] Zak DE (2016). A blood RNA signature for tuberculosis disease risk: A prospective cohort study. Lancet.

[13] WHO. *Tuberculosis, Leprosy and TB/HIV Prevention and Control Programme Manual Fourth Edition*. (2008).

[14] Gebremicael G (2019). Gene expression profiles classifying clinical stages of tuberculosis and monitoring treatment responses in Ethiopian HIV-negative and HIV-positive cohorts. PLoS ONE.

[15] Joosten SA (2012). Identification of biomarkers for tuberculosis disease using a novel dual-color RT-MLPA assay. Genes Immun..

[16] Geluk A (2014). Longitudinal immune responses and gene expression profiles in type 1 leprosy reactions. J. Clin. Immunol..

[17] WHO. *Nutritional Landscape Information System: Country Profile Indicators: Interpretation Guide*. (2010).

[18] Do H-T, Baars W, Borns K, Windhagen A, Schwinzer R (2006). The 77Câ†’G mutation in the human CD45 (*PTPRC*) gene leads to increased intensity of TCR signaling in T cell lines from healthy individuals and patients with multiple sclerosis. J. Immunol..

[19] Wang Y, Johnson P (2005). Expression of CD45 lacking the catalytic protein tyrosine phosphatase domain modulates Lck phosphorylation and T cell activation. J. Biol. Chem..

[20] Champagne P (2001). Skewed maturation of memory HIV-specific CD8 T lymphocytes. Nature.

[21] Ullum H (1997). Increased losses of CD4+CD45RA+ cells in late stages of HIV infection is related to increased risk of death: Evidence from a cohort of 347 HIV-infected individuals. AIDS (London, England).

[22] Miedema F (1992). Immunological abnormalities in the natural history of HIV infection: Mechanisms and clinical relevance. Immunodefic. Rev..

[23] Ullum H (1997). Increased losses of CD4+CD45RA+ cells in late stages of HIV infection is related to increased risk of death: evidence from a cohort of 347 HIV-infected individuals. AIDS.

[24] Choi BS, Park YK, Lee JS (2002). The CD28/HLA-DR expressions on CD4+T but not CD8+T cells are significant predictors for progression to AIDS. Clin. Exp. Immunol..

[25] Vidya Vijayan KK, Karthigeyan KP, Tripathi SP, Hanna LE (2017). Pathophysiology of CD4+ T-cell depletion in HIV-1 and HIV-2 infections. Front. Immunol..

[26] Brodeur J-F, Li S, Martins MDS, Larose L, Dave VP (2009). Critical and multiple roles for the CD3Îµ intracytoplasmic tail in double negative to double positive thymocyte differentiation. J. Immunol..

[27] Punt S (2015). Correlations between immune response and vascularization qRT-PCR gene expression clusters in squamous cervical cancer. Mol. Cancer.

[28] Hou Y, Zhang G (2021). Identification of immune-infiltrating cell-related biomarkers in hepatocellular carcinoma based on gene co-expression network analysis. Diagn. Pathol..

[29] Lundtoft C (2017). Aberrant plasma IL-7 and soluble IL-7 receptor levels indicate impaired T-cell response to IL-7 in human tuberculosis. PLoS Pathog..

[30] Peng Y (2017). Forced expression of IL-7R promotes CD8 T cell cytotoxicity to self antigen. PLoS One.

[31] Vesosky B, Rottinghaus EK, Stromberg P, Turner J, Beamer G (2010). CCL5 participates in early protection against *Mycobacterium tuberculosis*. J. Leukoc. Biol..

[32] Aldinucci D, Colombatti A (2014). The inflammatory chemokine CCL5 and cancer progression. Mediat. Inflamm..

[33] Lee J-S (2008). Depressed CCL5 expression in human pulmonary tuberculosis. JBV.

[34] Toossi Z (2001). Impact of tuberculosis (TB) on HIV-1 activity in dually infected patients. Clin. Exp. Immunol..

[35] Mekonnen E, Bekele E, Stein CM (2018). Novel polymorphisms in TICAM2 and NOD1 associated with tuberculosis progression phenotypes in Ethiopian populations. Glob. Health Epidemiol. Genom..

[36] Park JH (2007). Nod1/RICK and TLR signaling regulate chemokine and antimicrobial innate immune responses in mesothelial cells. J. Immunol..

[37] Frutuoso MS (2010). The pattern recognition receptors Nod1 and Nod2 account for neutrophil recruitment to the lungs of mice infected with Legionella pneumophila. Microbes Infect..

[38] Hall NB (2015). Polymorphisms in TICAM2 and IL1B are associated with TB. Genes Immun..

[39] Dheda K (2005). In vivo and in vitro studies of a novel cytokine, interleukin 4delta2, in pulmonary tuberculosis. Am. J. Respir. Crit. Care Med..

[40] Djoba Siawaya JF (2008). Differential expression of interleukin-4 (IL-4) and IL-4 delta 2 mRNA, but not transforming growth factor beta (TGF-beta), TGF-beta RII, Foxp3, gamma interferon, T-bet, or GATA-3 mRNA, in patients with fast and slow responses to antituberculosis treatment. Clin. Vaccine Immunol..

