# Supplementary material for: Low transcriptomic of PTPRCv1 and CD3E is an independent predictor of mortality in HIV and tuberculosis co-infected patient
Source: Sci Rep. 2022 Jun 16;12:10133. doi: 10.1038/s41598-022-14305-8 (PMC9203579; doi:10.1038/s41598-022-14305-8)
Supplement: Supplementary file 1 — Supplementary Tables. [file 41598_2022_14305_MOESM1_ESM.doc]

**Low transcriptomic of PTPRCv1 and CD3E is an independent predictor of mortality in HIV and tuberculosis co–infected Patient**

Gebremedhin Gebremicael, Atsbeha Gebreegziaxier and Desta Kassa

| **Supplementary Table 1. Baseline demographic and clinical characteristics of the study populations.** | | | |  |
| --- | --- | --- | --- | --- |
| **Characteristics** | **Death group (n=9)** | **Matched control (n=18)** | **P-value** |  |
| **Demographic data** |  |  |  |  |
| Age, years | 31.9 ± 8.9 | 32.4 ± 9.6 | 0.9382 |  |
| Female, n (%) | 3 (33.3) | 6 (33.3) | 1.000 |  |
| **Clinical data** |  |  |  |  |
| BMI, kg/m^2^ | 18.04 ± 3.2 | 19.32 ± 2.5 | 0.4250 |  |
| BMI < 18.50 kg/m^2^, n (%) | 4 (44.4) | 7 (38.9) | 0.658 |  |
| **Laboratory data** |  |  |  |  |
| CD4^+^ T cell count/µl (Total population) | 226.2 ± 265.7 | 234.7 ± 118.1 | 0.1985 |  |
| **HIV VL** |  |  |  |  |
| HIV VL (Log10) | 4.6 ± 0.9 | 4.2±1.3 | 0.5715 |  |
|  | | | | |

Data indicate means ± standard deviations (SD) unless stated otherwise. Significant differences between the study groups were determined using Wilcoxon Mann-Whitney test for continues variables and chi^2^ for the proportion of dichotomous variables. n (%): Number of patients (Percentage of patients); BMI: Body Mass Index; HIV VL: HIV plasma viral load

| **Supplementary Table 2. List of target genes for dcRT-MLPA.** | | | | |  |  |
| --- | --- | --- | --- | --- | --- | --- |
|  |  |  |  |  |  |  |
| **Immune cell subset markers** |  | **Treg associated genes** |  | **Chemokines** |  | **Inflammation** |
| CD19 |  | CCL4 |  | CCL13 |  | MMP9 |
| NCAM1 |  | CTLA4 |  | CCL19 |  | SPP1 |
| **T cell subset markers** |  | FOXP3 |  | **Pattern recognition receptors** |  | TIMP2 |
| CD3E |  | IL2RA |  | CD209 |  | **IFN signaling genes** |
| CD4 |  | LAG3 |  | CLEC7A |  | FCGR1A |
| CD8A |  | TGFB1 |  | MRC1 |  | **Cell Growth/proliferation** |
| CCR7 |  | TNFRSF18 |  | MRC2 |  | AREG |
| IL7R |  | **Cytotoxicity markers** |  | NOD1 |  | TGFBR2 |
| PTPRCv1 |  | GNLY |  | NOD2 |  | **Small GTPases/(Rho) GTPase activating proteins** |
| PTPRCv2 |  | GZMA |  | TLR1 |  | RAB13 |
| AIRE |  | GZMB |  | TLR2 |  | RAB24 |
| **Th1 associated genes** |  | PRF1 |  | TLR3 |  | RAB33A |
| CXCL10 |  | **Apoptosis/survival** |  | TLR4 |  | TAGAP |
| IFNG |  | CASP8 |  | TLR5 |  | TBC1D7 |
| IL1B |  | BCL2 |  | TLR6 |  | **Anti-microbial activity** |
| IL2 |  | FASLG |  | TLR7 |  | BPI |
| IL15 |  | FLCN1 |  | TLR8 |  | LTF |
| TBX21 |  | TNFRSF1A |  | TLR9 |  | **E3 ubiquitine protein ligase** |
| TNF |  | TNFRSF1B |  | TLR10 |  | NEDD4L |
| **Th2 associated genes** |  | **Myeloid associated genes** |  | **Inflammasome components** |  | **Scavenger receptor** |
| GATA3 |  | CD14 |  | NLRC4 |  | MARCO |
| IL4 |  | CD163 |  | NLRP1 |  | **Transcriptional regulators/activators** |
| IL4δ2 |  | CCL2 |  | NLRP2 |  | CAMTA1 |
| IL5 |  | CCL5 |  | NLRP3 |  | TWIST1 |
| IL6 |  | CCL22 |  | NLRP4 |  | ZNF331 |
| IL10 |  | CXCL13 |  | NLRP6 |  | ZNF532 |
| IL13 |  | IL12A |  | NLRP7 |  | **Intracellular transport** |
| **Th9 associated genes** |  | IL12B |  | NLRP10 |  | SEC14L1 |
| IL9 |  | IL23A |  | NLRP11 |  | **G-protein-couples receptors** |
| **Th17 associated genes** |  | FPR1 |  | NLRP12 |  | BLR1 |
| IL17A |  |  |  | NLRP13 |  | **Reference genes** |
| IL22RA1 |  |  |  |  |  | ABR |
| RORC |  |  |  |  |  | B2M |
|  |  |  |  |  |  | GAPDH |
|  |  |  |  |  |  | GUSB |

105 selected genes and 4 housekeeping genes to profile innate and adaptive immune responses.
